# Liability and Standard of Care in AI-Driven Psychiatric Practice: European Viewpoint

**DOI:** 10.2196/99894

**Published:** 2026-10-05

**Authors:** Luis Madeira, Cíntia Águas, Meryam Schouler-Ocak, Jerzy Samochowiec, Silvana Galderisi, Mariana Pinto da Costa

**Affiliations:** 1 Centre of Bioethics and Medical Humanities, College of Medicine, University of Lisbon, Lisbon, Portugal; 2 Department of Psychiatry, Hospital CUF Descobertas, Lisbon, Portugal; 3 Psychiatric University Clinic of Charité at St. Hedwig-Hospital, Charité - Universitätsmedizin Berlin, Berlin, Germany; 4 Department of Psychiatry, Pomeranian Medical University, Szczecin, West Pomerania, Poland; 5 Department of Mental and Physical Health and Preventive Medicine, University of Campania, Naples, Campania, Italy; 6 Institute of Psychiatry, Psychology & Neuroscience, King's College London, 16 De Crespigny Park, London, England, SE5 8AF, United Kingdom, 351 933 655 93; 7 South London and Maudsley NHS Foundation Trust, London, England, United Kingdom

**Keywords:** artificial intelligence, psychiatry, liability, legal, medical ethics, mental health, clinical decision support systems, digital health, data protection, standard of care

## Abstract

AI is increasingly incorporated into psychiatric triage, risk prediction, passive monitoring, clinical documentation, and patient-facing conversational systems. These applications may improve access, continuity, efficiency, and pattern recognition, but they also redistribute epistemic authority and complicate responsibility when harm occurs. European regulation is developed in relation to market access, data governance, risk management, and product safety, yet remains fragmented regarding civil liability, organizational negligence, and the psychiatric standard of care. This Viewpoint examines how liability and standard of care should be understood when AI becomes part of psychiatric reasoning in Europe. It advances one central thesis: psychiatric AI requires justified integration supported by layered accountability within, but not determined by, European regulation. It presents a targeted doctrinal and normative synthesis of binding European Union instruments, regulatory guidance, selected national governance materials, and psychiatric, bioethical, legal, and digital mental health literature. It distinguishes binding law from guidance and policy, and separates ex ante regulation from ex post liability, and from professional standards of care. Four illustrative domains are analyzed: conversational or therapeutic chatbots, suicide prediction, digital phenotyping and passive monitoring, and large language model documentation. Psychiatric AI raises distinctive concerns because psychiatric judgment depends heavily on testimony, contextual meaning, therapeutic trust, risk interpretation, privacy, and liberty-sensitive decisions. Existing European instruments, including the AI Act, Medical Device Regulation, General Data Protection Regulation, revised Product Liability Directive, and European Health Data Space Regulation, establish governance duties, but do not provide a harmonized fault-based liability framework for AI-assisted health care. Regulatory compliance may inform later legal assessment, but it does not determine whether psychiatric care was reasonable. The proposed standard of justified integration requires knowledge of intended use and model limits, assessment of local and patient-level applicability, active clinical interpretation, disclosure when AI use is material to consent or trust, documentation in high-stakes decisions, and organizational audit. Accountability should be distributed across developers, deployers, and clinicians according to control and preventability. Mixed-fault scenarios are therefore likely to be common. The augmented-clinician model and layered accountability are offered as normative proposals rather than settled European legal standards. Clinicians should remain responsible for contextual, patient-centered judgment; developers for design, validation, documentation, and foreseeable misuse; and deployers for procurement, training, workflow integration, local validation, monitoring, and escalation. Future empirical research should evaluate effects on clinician reliance, documentation burden, patient outcomes, coercive interventions, therapeutic trust, and feasibility across differently resourced services.

## Introduction

AI is no longer peripheral to psychiatric practice. Across Europe, AI systems are being developed for diagnostic support, triage, risk prediction, digital phenotyping, psychotherapy adjuncts, clinical documentation, and patient-facing conversational interfaces. Their appeal is understandable. Psychiatry faces shortages of professionals, uneven geographical access, long waiting times, variable continuity of care, and decisions often made under uncertainty, incomplete data, and time pressure. AI may support faster pattern recognition, longitudinal monitoring, automated documentation, and more consistent triage. It is also often presented as a tool for equity, since AI-supported services may extend their reach to underserved populations. Yet, these benefits are conditional: poorly designed or poorly deployed systems may reproduce, conceal, or intensify existing disparities in mental health care [1-5].

Psychiatry is not simply another data-rich specialty to which AI can be straightforwardly applied, as its clinical object is often reported experience, relational style, behavior, temporality, and evolving narrative, rather than a stable biomarker. Diagnosis and treatment depend on interpretation of meaning, context, vulnerability, and trust, which makes the legal consequences of adopting AI systems or models in this field especially complex. In psychiatry, an AI output may influence whether a patient is believed, whether distress is treated as risk, whether coercion is initiated, or whether a simulated therapeutic conversation is mistaken for meaningful care. The resulting harms may be clinical, relational, epistemic, dignitary, or psychological, and several actors may contribute to them simultaneously [6-9]. The central legal question is therefore not only whether AI is accurate, but how responsibility should be allocated when AI becomes entangled with psychiatric judgment. If a suicide-risk model underestimates imminent danger, liability may concern the clinician who relied on it, the institution that integrated it, or the developer that trained it on biased or impoverished data. If a therapeutic chatbot fosters dependence, validates delusional beliefs, or mishandles crisis disclosures, the relevant framework may involve medical negligence, product defect, consumer protection, data protection, or a hybrid of these. Conversely, if a psychiatrist departs from an algorithmic recommendation and harm follows, courts may later need to decide whether that departure was prudent clinical judgment or negligent disregard of an available tool. AI may improve access, fairness, and efficiency, but it may also create new vulnerabilities where apparently therapeutic systems simulate empathy without corresponding responsibility [10-16].

The current European framework rests on overlapping instruments. The Medical Device Regulation (MDR) governs many clinical software tools, including software intended for diagnosis, prediction, or therapeutic support, with Medical Device Coordination Group (MDCG) guidance addressing medical device software and AI [17,18]. The General Data Protection Regulation (GDPR) structures the processing of personal and special-category health data. The AI Act establishes a risk-based regime in which many health care apps are high-risk, and certain manipulative or exploitative uses are prohibited [15,19]. The revised Product Liability Directive extends strict liability to software and AI-enabled products, while recognizing medically cognizable psychological harm and, in defined cases, compensable loss or corruption of data [16]. The European Health Data Space (EHDS) may also become relevant where high-risk AI systems interact with electronic health records.

Although the AI Act establishes an important regulatory framework, it is primarily preventive rather than compensatory. It imposes obligations on providers and deployers but does not create a civil liability regime for harm after it occurs. The revised Product Liability Directive modernizes strict product liability; yet, it remains focused on defectiveness rather than the broader spectrum of negligent clinical reliance, organizational misuse, or inadequate local implementation. The European Commission proposed an AI Liability Directive to ease causation and disclosure burdens in fault-based claims, but its 2025 work program listed the proposal for withdrawal because no foreseeable agreement could be reached [20]. Consequently, Europe has a relatively sophisticated compliance regime for AI, but no harmonized fault-based civil liability regime tailored to AI-assisted health care. Patients and clinicians in psychiatric services remain dependent on EU (European Union) product law, national malpractice doctrines, contractual arrangements, and professional guidance [14,20].

This Viewpoint advances a practical argument for AI-assisted psychiatric care in Europe. Its aim is to clarify how liability and standard of care should be understood when AI becomes part of psychiatric reasoning, without treating AI either as an autonomous substitute for the clinician or as a neutral instrument whose risks remain solely with the user. It is intended for psychiatrists and other mental health clinicians, health care organizations, AI developers and vendors, professional bodies, lawyers, regulators, policymakers, and insurers involved in AI adoption or governance in mental health care.

It advances four linked messages that are components of one central claim: psychiatric AI requires what we term justified integration, namely the reasoned incorporation of AI into clinical judgment through critical interpretation rather than automatic acceptance or rejection. First, psychiatric AI should be assessed through a standard of justified integration, requiring clinicians to understand when an AI output is relevant, when it should be followed, and when it should be overridden. Second, European AI regulation should be distinguished from civil liability and from the psychiatric standard of care, since regulatory compliance may mandate requirements that must be met, but it does not determine whether the care provided was reasonable. Third, psychiatric AI raises specific risks because it may affect credibility, coercion, documentation, privacy, therapeutic trust, and the interpretation of suffering. Fourth, responsibility should be layered across developers, deployers, and clinicians, since harm may arise from design, procurement, workflow, training, validation, or clinical use. The doctrinal component identifies the existing legal constraints and gaps; the normative component explains how professional and institutional practice should respond to them. This Viewpoint is informed by clinical psychiatric practice, European professional and academic experience in mental health ethics, and a targeted doctrinal and normative synthesis of legal, policy, psychiatric, bioethical, and digital mental health literature. It is not presented as a systematic review, empirical study, or comprehensive comparative legal analysis. Figure 1 summarizes these key messages.

**Figure 1.**
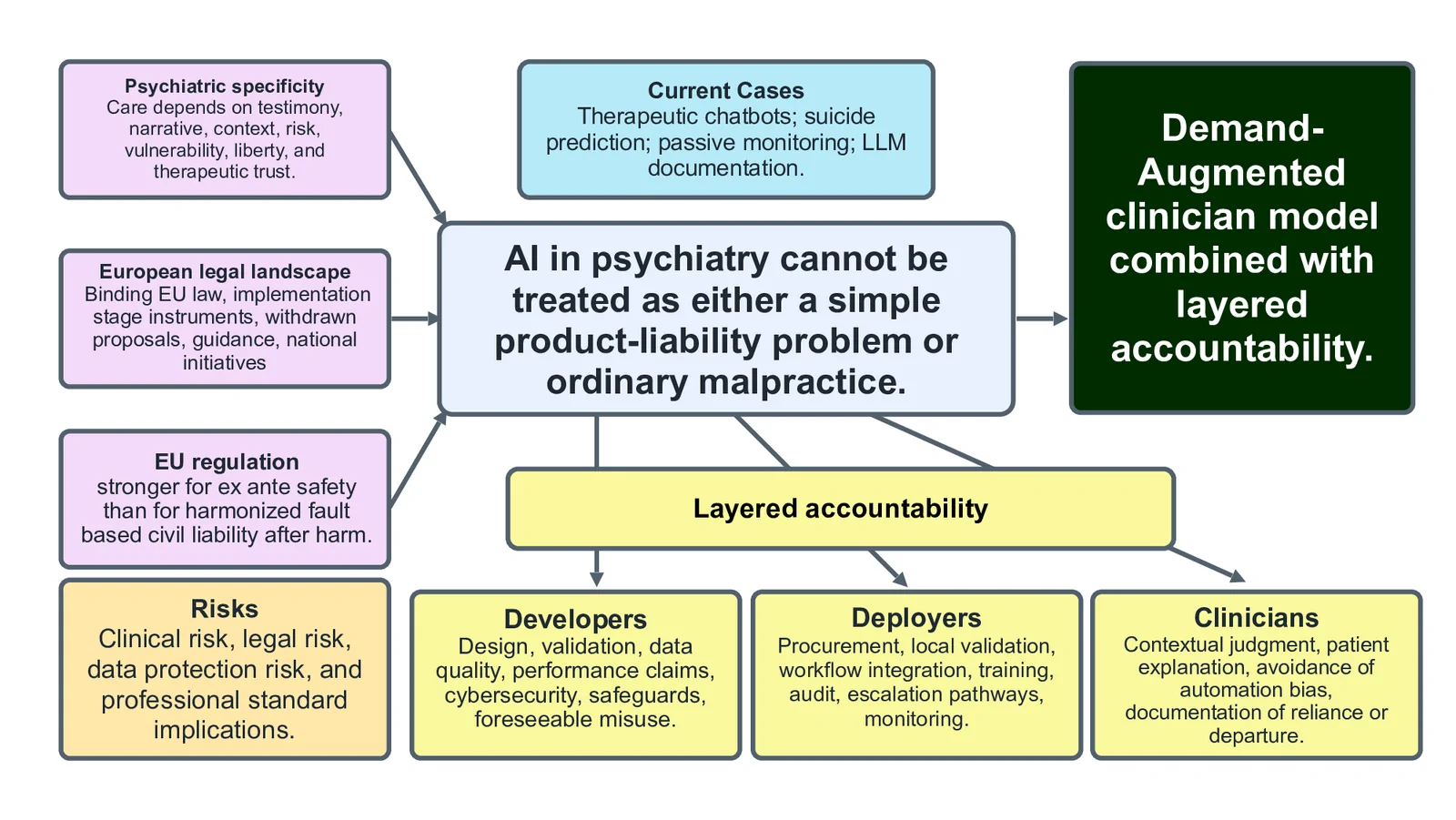
Liability and standard of care in AI-driven psychiatric practice. LLM: large language model.

## Key Message 1: Psychiatric AI Changes Liability Before Litigation

### Why Psychiatry Changes the Liability Analysis

Liability analysis in health care AI often begins with a general question: if an AI system contributes to harm, should responsibility attach to the manufacturer, the deployer, the clinician, or some combination of these actors? This framing is useful, but it overlooks what is distinctive about psychiatry. In this article, “psychiatric AI” is shorthand for AI systems whose outputs or interactions materially influence psychiatric assessment, treatment, monitoring, documentation, or patient support; it does not imply that the underlying technology is unique to psychiatry. Many risks are shared with health care AI, but they may be intensified in psychiatry because decisions depend unusually heavily on testimony, contextual interpretation, therapeutic trust, and liberty-sensitive risk judgments. Psychiatric practice depends on first-person reports, family testimony, behavioral signs, temporality, and shifting contextual meaning. The clinician’s task is rarely binary detection; it involves integrating narrative, risk, diagnosis, and relationship in a setting where the patient may be frightened, ambivalent, cognitively impaired, socially vulnerable, or legally exposed. AI therefore alters not only efficiency, but also the epistemic structure of the encounter [1,6-8]. For this reason, the standard of care in psychiatry cannot be reformulated merely in terms of improved predictive accuracy. It must remain grounded in justified clinical reasoning. Muyskens et al [21] argue that epistemic humility in clinical practice requires recognition of uncertainty, awareness of the limits of knowledge, and respectful engagement with other epistemic sources, including the patient. In computational psychiatry, this is crucial. If algorithmic output is treated as inherently superior because it appears quantitative or objective, the clinical encounter risks becoming a form of machine-credentialed disbelief. A patient’s account may then be displaced because it is inconsistent with a model’s score or category. In psychiatry, where patient testimony is often the primary source of knowledge, this is a structural threat to good practice [1,21].

This problem can be described as testimonial injustice in a digital context. AI does not create testimonial injustice, which can arise from ordinary clinical power asymmetries, but it may amplify and institutionalize it by attaching quantitative authority to an opaque output and reproducing that judgment across clinical workflows. A patient reporting escalating suicidal thoughts, newly commanding hallucinations, or intolerable medication effects may be implicitly disregarded when an AI system labels risk as low or adherence as satisfactory. Once institutions present such systems as validated, clinicians may feel legally, professionally, and institutionally exposed if they depart from the tool without documenting why, but equally exposed if they follow it and harm results. Liability pressure therefore does not arise only after error; it shapes the distribution of authority before decisions are made, including in triage, detention, discharge, and therapeutic alliance [2,14,22,23].

This prelitigation effect is supported by empirical work on psychiatric defensive practice. In an Italian national survey of 254 psychiatrists, most respondents reported defensive medical behaviors in the context of perceived professional liability and medicolegal pressure, a concern sharpened in Italy by the medicolegal position of guarantee, under which psychiatrists may be held responsible not only for diagnostic and therapeutic appropriateness but also for some consequences of patient behavior [24]. Additional analysis from the same survey found that defensive practice was associated with a group that is younger in age, had fewer years of professional experience, and female, while prior involvement in complaints was not the main differentiating factor [25]. These findings do not establish a Europe-wide pattern, but they support the psychiatric claim that liability pressure may alter ordinary clinical reasoning before litigation occurs, particularly in decisions about admission, discharge, risk assessment, compulsory care, and documentation.

### From Automation Bias to Human-in-Reasoning

The legal literature often assumes that human oversight resolves accountability. The AI Act similarly requires meaningful human oversight for high-risk systems [15]. In practice, formal oversight does not guarantee substantive independence. Work on automation bias and selective adherence shows that human decision-makers tend to over-rely on automated outputs when systems appear technically advanced, carry institutional endorsement, or operate in time-pressured settings [26]. Direct psychiatry-specific evidence remains limited, but general automation-bias research and the defensive-practice literature discussed above suggest that uncertainty and concerns about defensibility may intensify deference to automated outputs. A clinician faced with a probabilistic risk score or large language model (LLM) summary may outsource doubt to the machine. While AI promises consistency, opacity surrounding model development, validation, and domain transfer can make challenging its conclusions difficult. Psychiatric professionals may remain formally responsible while becoming practically deferential, creating a misalignment in which liability remains human while practical epistemic authority shifts toward the system [14,27-29]. Here, authority means the weight given to the output in reasoning, not a legal transfer of decision-making power. Reliance may be more difficult to evaluate than reliance on a validated scale or a colleague’s advice when the model is opaque, dynamically updated, institutionally mandated, or difficult to challenge. The appropriate response is not a symbolic human-in-the-loop requirement, but a standard of human-in-reasoning: the substantive work of understanding the output’s intended use and limits, testing it against the patient and context, and recording reasons for reliance or departure. Clinicians must be able to interpret outputs sufficiently to assess their fit with the patient, context, and legal stakes. Institutions should require active articulation of reasons when clinicians follow or override high-stakes AI outputs, especially in admission, discharge, compulsory care, and suicide-risk assessment. AI explainability need not entail full technical transparency, but psychiatric systems should provide intelligible reasoning, confidence limits, performance boundaries, and warnings when extrapolating from weakly matched data; otherwise, clinicians may not have sufficient or adequate outputs to support interpretation [4,15,28,30,31].

## Key Message 2: European Regulation Does Not Resolve Civil Liability

The normative materials discussed in this section do not have the same legal force. The AI Act, the MDR, the GDPR, the revised Product Liability Directive, and the EHDS Regulation are binding EU instruments, although their practical effects differ, as regulations are directly applicable while directives require national transposition. The AI Act and the EHDS Regulation also apply progressively, so some obligations may be legally adopted but not yet fully operative in clinical practice. The proposed AI Liability Directive is treated differently: it was listed for withdrawal in the European Commission’s 2025 work program and is not analyzed as current law, but as evidence that harmonized EU fault-based liability for AI remains absent. MDCG documents and European Commission guidelines are discussed as regulatory guidance that may influence interpretation and compliance but should not be equated with civil liability rules. National AI strategies, sandboxes, reimbursement frameworks, and institutional webpages are used only as illustrative governance materials. Ethical and professional literature supports the normative argument about psychiatric standard of care, but it does not itself establish legal duties.

The European framework is strongest in ex ante regulation. The AI Act entered into force on August 1, 2024, and applies progressively: since February 2, 2025, rules prohibiting unacceptable AI practices and requiring AI literacy have applied; since August 2, 2025, governance provisions and obligations for general-purpose AI have applied; and the main obligations for high-risk systems apply from August 2, 2026, with longer transition periods for some AI embedded in regulated products. Under EU harmonization law, AI systems used as safety components of regulated products, or as regulated products themselves, including medical devices, are generally classified as high-risk. Psychiatric clinical software may therefore become legally significant before negligence litigation arises [15,18].

The AI Act matters for psychiatry because it imposes design and governance duties on high-risk systems, including risk management, data governance, technical documentation, logging, transparency to deployers, and human oversight. It foregrounds bias and dataset representativeness, which is central in mental health care because diagnostic labels, service-use patterns, and coercion histories may reflect social inequalities rather than stable clinical realities. It also prohibits manipulative or exploitative practices, including systems that exploit vulnerabilities related to age, disability, or social or economic situation in ways that materially distort behavior and cause significant harm. The Commission’s 2025 guidelines interpret these prohibitions broadly enough to include risks associated with mental health chatbots and similar systems [19]. The Act provides a shared regulatory vocabulary that courts may later use when assessing reasonable deployment or defective design, although it does not harmonize malpractice law.

The revised Product Liability Directive narrows part of the compensation gap by modernizing EU product liability for the digital age. It covers software and AI-enabled products and recognizes medically recognized psychological harm as compensable damage [16]. It also permits proportionate disclosure of relevant evidence where a claimant has presented sufficient facts to support the plausibility of a claim. In defined circumstances, defectiveness may be presumed when relevant evidence is not disclosed or when technical complexity makes proof excessively difficult, and a causal link may be presumed when established defectiveness is of a kind likely to have caused the damage [16]. These mechanisms reduce, but do not remove, the claimant’s burden of proving damage, defect, and causation. This is important for psychiatry, since mental health harms have often fitted poorly within liability frameworks centered on physical injury and tangible goods. Yet, product liability does not solve the whole problem. Many psychiatric harms will not be reducible to product defect. A technically compliant tool may be used in an organizationally negligent way; a hospital may deploy a triage model without training, escalation pathways, or local validation; and a chatbot may sit ambiguously between wellness software and medical functionality. These fault-based questions exceed strict product liability. The nonadoption of the proposed AI Liability Directive is therefore consequential, even more so if AI models and systems in use are developed outside the EU. The proposal was intended to assist fault-based claims through disclosure of relevant evidence and a rebuttable presumption of causality where a relevant duty was breached and that breach was reasonably likely to have influenced the AI output and resulting damage. The European Commission’s 2025 work program listed the proposal on adapting noncontractual civil liability rules to AI for withdrawal because no foreseeable agreement could be reached [20]. The EU therefore lacks a harmonized AI-specific negligence framework, leaving psychiatric claimants dependent on national tort law, contract law, professional liability regimes, and variable evidentiary standards. In opaque mental health systems, this leaves a major proof burden in place.

It is important to distinguish regulation, liability, and clinical standard of care. Regulatory compliance may be relevant evidence when courts assess reasonable conduct, organizational negligence, or product defect, but it is not identical to the psychiatric standard of care. A system may comply with the AI Act or MDR and still be used negligently in a particular service. Conversely, a clinician may satisfy the psychiatric standard of care by declining or overriding a compliant tool when its output does not fit the patient, the setting, or the liberty-sensitive stakes of the decision. Product liability concerns defectiveness of software or AI-enabled products; organizational negligence concerns procurement, local validation, training, workflow, monitoring, and escalation; and individual malpractice concerns whether the psychiatrist reasoned and acted in a manner consistent with competent psychiatric practice in the circumstances. These categories may overlap, but they should not be collapsed.

Several tools occupy a regulatory gray zone. Here, “gray zone” does not mean that no regulation applies; it refers to uncertainty over product classification, intended purpose, and the applicable duties when tools marketed as wellness or administrative software materially influence clinical care. Journaling apps with sentiment analysis may claim wellness benefits; chatbots may advertise resilience or self-help rather than treatment; and documentation assistants may influence care by reframing records without presenting themselves as diagnostic tools. Formal classification alone cannot determine the standard of care; courts and regulators must consider functional use, foreseeable reliance, and user vulnerability [13,23,32-34]. Some psychiatric AI tools will clearly fall within MDR, including software intended to predict relapse, support diagnosis, recommend treatment, or clinically monitor patients under the MDR and MDCG guidance, in which case conformity assessment, postmarket surveillance, and clinical evaluation strengthen later liability analysis [17,18]. The GDPR is relevant beyond privacy. Psychiatric AI systems often process special-category health data, infer mental states, profile risk, and support consequential decisions[35]. Defective data governance may contribute directly to harm when incomplete, unrepresentative, poorly curated, or repurposed data generate biased outputs that shape triage, diagnosis, discharge, or coercive intervention. The EHDS Regulation may add complexity by strengthening cross-border access to digital health records [36], which in the case of psychiatry might include digitized therapy notes and risks of breach, reidentification, stigma, and discriminatory use of pseudonymized mental health data. Secondary use through opt-out mechanisms raises concerns about privacy, autonomy, transparency, and commercial access to sensitive behavioral and health information. Psychiatric AI therefore requires strict rules on how mental health data are accessed, reused, and used to train models.

## Key Message 3: Accountability Should Be Layered

### From Individual Liability to Layered Accountability

Responsibility refers to the substantive duties and causal contributions of actors; accountability refers to the mechanisms through which they must explain, document, audit, and answer for those duties; and liability refers to the legal consequences and remedies that may follow after harm. The terms overlap but are not interchangeable. Theoretical accounts of AI liability in medicine often distinguish between 3 models. Under a reliance model, clinicians are encouraged to rely on certified systems and may be partly shielded when doing so reasonably. Under a deviance model, clinicians remain fully liable because AI is treated as a tool. Under a neutrality or no-fault model, compensation is socialized through insurance or collective funds, reducing the plaintiff’s burden to prove individual fault [14]. Each model captures an important dimension, but none is sufficient for psychiatry. A pure reliance model risks amplifying automation bias and diminishing the clinician’s duty to interpret the patient. A pure deviance model places disproportionate responsibility on psychiatrists while allowing developers and institutions to externalize risks associated with opaque systems they control. A pure neutrality model may improve access to compensation but risks underdeterrence unless combined with robust regulatory enforcement and recourse mechanisms. At present, claimants may face substantial costs in overcoming opacity and producing evidence of wrongdoing, breach of duty, causation, and harm. Psychiatric practice therefore calls for a layered model of accountability and a specific liability framework for AI.

Responsibilities are allocated according to the degree of control over the relevant source of risk and the capacity to prevent or mitigate foreseeable harm. Actors should primarily be accountable for aspects of AI-assisted care that they design, introduce, govern, or directly influence. This functional allocation reflects well-established principles in product liability, organizational governance, and professional negligence, while recognizing that psychiatric AI operates through sociotechnical systems rather than isolated individual decisions. Responsibilities therefore follow control and preventability rather than professional status alone. Developers, as they control system architecture, validation, documentation, intended use, and foreseeable misuse, should be responsible for design defects, inadequate validation, misleading performance claims, cybersecurity failures, and insufficient safeguards against foreseeable misuse. Deployers, typically health care organizations or institutions responsible for procuring and implementing AI systems, should be accountable for procurement, local validation, workflow integration, staff training, monitoring, and escalation procedures because they determine how the technology enters and functions within clinical services. Clinicians should remain responsible for informed, context-sensitive judgment, avoiding uncritical reliance, and documenting reasons in high-stakes cases because they retain immediate responsibility for applying AI outputs to an individual patient within a particular clinical context [27,30]. Table 1 proposes the main liability models in health care AI and their psychiatric implications.

**Table 1.** Main liability models in health care AI and their psychiatric implications^a^.

Liability model	Core legal logic	Main psychiatric implication
Reliance model	Reasonable reliance by a clinician on a validated AI system may reduce or partially shift individual fault exposure.	Risks encouraging overreliance on algorithmic outputs, especially in triage and suicide-risk assessment, unless accompanied by explainability, active documentation, and careful oversight.
Deviance model	The clinician remains fully responsible because AI functions as a tool within professional judgment.	Preserves clear human accountability, but may incentivize defensive practice and place disproportionate risk on clinicians where systems are opaque or institutionally mandated.
Layered accountability model	Responsibility is distributed across developers, deployers, and clinicians according to the level at which failure occurs.	Better reflects psychiatric care, where harms often arise from interactions between product design, workflow, and contextual clinical reasoning.
Neutrality or no-fault model	Compensation is socialized through insurance, compensation funds, or similar mechanisms, reducing the claimant’s need to prove individual fault.	May improve access to compensation after AI-related psychiatric harm, but risks underdeterrence unless combined with regulatory enforcement, audit, and recourse against developers or deployers.

a Adapted from Bottomley and Thaldar [14] and developed for psychiatric use in this manuscript.

### Illustrative European Governance Trajectories

Because fault-based liability is largely governed at the national level, the European legal and practical landscape remains uneven. The following examples are an illustrative mapping of different governance trajectories among countries represented by the authors or commonly discussed in European digital health governance: strategy-led implementation, centralized supervision, human-primacy legislation, evidence-based reimbursement, guidance-led trust governance, and sandbox preparation. Portugal illustrates a strategy-led approach. The country approved a National AI Agenda for 2026 to 2030 through Council of Ministers Resolution No. 2/2026 of January 8, but has not enacted a comprehensive standalone AI liability regime. The existing Center for Responsible AI has no regulatory or oversight capacity. Neither the Charter of Human Rights in the Digital Era nor the designation of ANACOM (Autoridade Nacional de Comunicações) as the single point of contact for the EU AI Act has addressed AI liability in psychiatric care. In health care, this means continued reliance on general health law, tort law, data protection law, medical device law, labor law, and incoming EU obligations. This offers flexibility but limited certainty in psychiatric malpractice scenarios involving AI-assisted care [37]. Spain has moved earlier toward institutional supervision through AESIA (Spanish Agency for the Supervision of AI), created under Royal Decree 729/2023 (August 22, 2023). This may support administrative oversight and coherent implementation of EU requirements, but ordinary negligence law must still determine reasonableness when clinicians follow, ignore, or depart from AI recommendations [38,39]. Italy has adopted one of the clearest human-primacy positions. Law No. 132/2025 (September 23, 2025) frames AI in health care as auxiliary rather than autonomous, prohibits discriminatory conditioning of access to health care, and affirms human responsibility for clinical decision-making. For psychiatry, this resists attributing autonomous clinical authority to machines, but may leave clinicians carrying extensive responsibility unless recourse against defective systems or negligent deployment is available.

Germany offers a different model through the Digitale Gesundheitsanwendungen framework. Digital health applications may be reimbursed under statutory insurance when they meet regulatory and evidentiary criteria, including demonstration of a positive health care effect. Although Digitale Gesundheitsanwendungen does not primarily address AI liability, it shows how evidence, reimbursement, and regulation can shape the practical standard of care [40]. France emphasizes governance and trust through the Haute Autorité de Santé *Initial Key Guiding Principles for the Use of Generative AI in Health Care* and the Health Data Hub, stressing appropriate use, verification, and organizational responsibility [41,42]. The Netherlands illustrates supervisory preparation of the AI Act implementation through sandbox approaches and continued reliance on civil code doctrines. These trajectories show convergence on governance but divergence on civil liability. For psychiatry, the same AI-supported act may face similar ex ante duties across Europe but different ex post litigation risks. Table 2 shows selected European jurisdictions toward AI governance.

**Table 2.** Illustrative mapping of selected European psychiatric AI governance trajectories^a^.

Jurisdiction	Illustrative legal or policy anchor	Institutional emphasis	Psychiatric relevance
Portugal	National AI Agenda 2026‐2030; general health, labor, and data protection framework	Strategy-led implementation within EU^b^ law	Psychiatric AI may expand before introduction of a dedicated national liability regime.
Spain	Royal Decree 729/2023 and AESIA^c^	Centralized supervisory architecture	Administrative oversight may improve, but ordinary tort law remains central after harm.
Italy	Law No. 132/2025	Strong human primacy in health care AI	Supports the view that psychiatric decision-making cannot be delegated to autonomous systems.
Germany	DiGA^d^ reimbursement and BfArM^e^ evaluation	Evidence-based reimbursement governance	Validated digital tools may shape expectations regarding reasonable clinical use.
France	HAS^f^ guidance on generative AI in health care; Health Data Hub strategy	Trust, governance, and organizational use	Prioritizes safe adoption rather than technical performance alone.
The Netherlands	AI Act sandbox preparation and civil code continuity	Compliance experimentation with existing liability law	Highlights reliance on general negligence and contractual doctrines.

a AESIA [38], BfArM [40], HAS [41], Portugal Global [37], and selected national legal or policy materials discussed in the text. This table is illustrative and should not be read as a structured comparative liability analysis.

b EU: European Union.

c AESIA: Spanish Agency for the Supervision of AI.

d DiGA: Digitale Gesundheitsanwendungen.

e BfArM: federal institute for drugs and medical devices.

f HAS: Haute Autorité de Santé.

### Four Psychiatric AI Domains That Test Liability Law

The following 4 use cases are illustrative rather than exhaustive. They were selected to represent common or rapidly expanding psychiatric AI domains. Each tests a different legal category: simulated therapeutic interaction, risk prediction, data-driven surveillance, and clinical record production. These technologies should not be treated as a single regulatory class. Patient-facing conversational agents may operate with limited clinician mediation; suicide-prediction systems function as clinical decision support; passive monitoring produces continuous inferential data; and documentation assistants are primarily administrative tools with indirect but potentially important clinical effects. Their intended purpose, degree of autonomy, and position in the workflow affect classification and liability exposure. The first is the conversational or therapeutic chatbot. Responsible AI principles focused solely on fairness, autonomy, and robustness may be insufficient when the clinical issue is relational: dependency, vulnerability, emotional attunement, and meanings generated through interaction must also be considered [23]. Chatbots may appear empathic and continuous without clinical responsibility or capacity for care. Reviews show rapid expansion, heterogeneous purposes, variable evidence, and uneven safeguards, while recent analyses warn about role confusion, dependence, and mismatches between the perception of empathy and accountable care [11,12,32,43,44]. When systems approximate therapeutic dialogue, crisis response, or management of vulnerable users, some duty of care becomes harder to deny [10,13,19,34]. The second use case is suicide prediction. AI-based models may outperform unaided clinical judgment in some settings, but reviews emphasize heterogeneity and difficulty translating prediction into ethical intervention [45]. Studies using electronic health records and social media content illustrate improved stratification, but also show how prediction can become detached from actionability, liberty, and fairness [46-49]. False negatives may produce underintervention; false positives may increase coercion, stigma, and deprivation of liberty. Risk scores should not become warrants for restriction [45,50]. The third use case is digital phenotyping and passive monitoring. Systems that infer mood, relapse, cognition, or adherence from smartphones, wearables, language, or behavioral traces rely on data that may be noisy, socially patterned, or weakly contextualized. Patients may not understand the extent of inference, and services may lack capacity to respond to alerts. Reviews stress the gap between proof-of-concept performance and safe clinical translation [3,6,8]. The fourth use case is LLM documentation and summarization. Psychiatric records shape continuity of care, risk assessment, legal processes, insurance decisions, and patients’ self-understanding. If AI compresses nuance, overstates certainty, omits qualifiers, or translates narratives into confident diagnostic shorthand, downstream care may be distorted. These systems should be evaluated for documentation fidelity, calibration, and downstream effects, not only fluency or time saving [28,29,51]. When appropriately validated and integrated, these tools may improve access, identify clinically relevant patterns, support continuity, and reduce documentation burden. The purpose of the liability analysis is to enable those benefits through safer use rather than to argue against adoption. Table 3 shows psychiatric AI use cases, liability risks, and standard-of-care implications.

**Table 3.** Psychiatric AI use cases, liability risks, and standard-of-care implications.

Use case	Clinical risk	Legal risk	Data protection risk	Professional standard implication	References
Conversational or therapeutic chatbots	Therapeutic simulation, role confusion, dependence, crisis mismanagement.	Ambiguous classification as wellness or health care software; foreseeable therapeutic reliance may create duties of care.	Sensitive disclosures may be captured, stored, reused, or insufficiently escalated in crisis contexts.	Require transparency, limits, crisis escalation, and safeguards against exploitative or manipulative use.	[10-13,19,23,32,34,43,44]
Suicide prediction	False negatives may lead to underintervention; false positives may lead to coercion, stigma, and deprivation of liberty.	Liability may arise from defective design, negligent deployment, or uncritical clinical reliance.	EHR^a^, language, and social media data may involve profiling, inference, and repurposing of special-category data.	Use scores as decision support, not warrants for restriction; require actionability, fairness assessment, and documented reasoning.	[45-50]
Digital phenotyping and passive monitoring	Noisy, socially patterned, or weakly contextualized data may generate unreliable alerts or surveillance burdens.	Responsibility may turn on local validation, alert management, response capacity, and escalation protocols.	Continuous behavioral monitoring increases risks of opacity, reidentification, function creep, and patient misunderstanding.	Clarify what is measured, review frequency, alert responsibility, patient comprehension, and escalation thresholds.	[3,6,8]
LLM^b^ documentation and summarization	Compression of nuance, overstated certainty, omitted qualifiers, and distorted downstream records.	Errors may support malpractice claims when records influence continuity, risk assessment, insurance, or legal processes.	Records may include inferred mental states, third-party information, and sensitive narrative material.	Evaluate documentation fidelity, calibration, downstream effects, and clinician verification duties.	[28,29,51]
Cross-cutting standard of care	Failures may arise from design, procurement, validation, workflow, training, clinical use, or monitoring.	Mixed-fault cases require separation of developer, deployer, and clinician responsibilities.	Data quality, representativeness, secondary use, access control, and auditability are central to safety.	Adopt justified integration and layered accountability; assess subgroup performance, comprehension, coercive effects, and trust.	[3,4,6,15,27,29,30,43,44]

a EHR: electronic health record.

b LLM: large language model.

## Key Message 4: The Standard of Care Should Be Justified Integration

### Operationalizing Justified Integration in Psychiatric Practice

Justified integration refers to the reasoned incorporation of AI into psychiatric decision-making through critical clinical interpretation rather than routine acceptance or automatic rejection of algorithmic outputs. We adopt the Integrated Ethical Approach for Computational Psychiatry proposed by Putica et al [27] because it is specifically designed for mental health AI and integrates the principal ethical domains that are repeatedly implicated in psychiatric AI, namely beneficence, autonomy, justice, privacy, transparency, and scientific integrity. These principles provide the ethical rationale for justified integration while remaining distinct from legal duties. In this Viewpoint, it supports the ethical content of justified integration but is not treated as a source of European legal duties. AI should inform psychiatric reasoning without replacing it. The standard of care should neither require routine acceptance of AI outputs nor treat AI use as inherently suspect. Under justified integration, psychiatrists should know the intended purpose and limits of the system, assess whether the patient resembles the population on which the tool was developed, consider whether the output fits the clinical picture, discuss AI involvement when material to consent or trust, and document reasons when high-stakes decisions rely on or depart from AI outputs. This approach aligns the augmented clinician model with the AI Act’s emphasis on meaningful human oversight, while translating oversight into a clinically operational duty [4,15,29,30]. Table 4 relates the clinical meaning of requirements for the reasoned integration of AI in psychiatric practice.

**Table 4.** Practical requirements of justified integration of AI in psychiatry.

Requirement	Clinical meaning	Primary responsibility
Intended use	The clinician and service know what the system is designed to do, what it is not designed to do, and whether it is diagnostic, predictive, administrative, or conversational.	Developer and deployer
Model limits	Users understand performance boundaries, uncertainty, subgroup limitations, and warnings about out-of-distribution use.	Developer and deployer
Local validation	The institution assesses whether performance and workflow are acceptable in the local population and service context before routine use.	Deployer
Patient fit	The psychiatrist considers whether the patient resembles the population and context for which the system was developed and validated.	Clinician
Clinical coherence	The AI output is checked against history, mental state examination, collateral information, risk formulation, and therapeutic context.	Clinician
Consent and transparency	AI involvement is disclosed when it is material to consent, trust, data use, or the patient’s understanding of care.	Deployer and clinician
High-stakes documentation	Reasons for following or overriding AI output are recorded in decisions involving suicide risk, admission, discharge, compulsory care, medication, or crisis escalation.	Clinician
Audit and feedback	The organization monitors performance, bias, alert fatigue, coercive effects, complaints, and downstream record quality.	Deployer

A further implication concerns the nature of evidence. Evidence for AI should not be limited to aggregate predictive accuracy; it should include subgroup performance, false positives and false negatives, user comprehension, impact on clinician behavior, impact on coercive interventions, and effects on therapeutic trust. A suicide-prediction tool that modestly improves discrimination but increases unnecessary coercion in specific populations may be clinically and legally inferior to a less accurate model deployed more cautiously. Likewise, an apparently effective chatbot may perform well on symptom reduction while generating role confusion, emotional overdependence, or unsafe crisis responses. Although European law does not yet mandate all such metrics, professional guidance and institutional governance should increasingly consider them when defining reasonable psychiatric use [29,43,44]. Implementation must be proportionate to clinical risk and workflow. Low-risk administrative tools may require verification and periodic audit, whereas systems influencing diagnosis, suicide risk, admission, discharge, or compulsory care require formal training, local validation, escalation procedures, and protected time for review. Institutions should avoid converting human oversight into unresourced documentation: concise templates, audit sampling, and clear ownership of alerts can reduce burden. Smaller services may require shared procurement expertise, regional validation support, or phased adoption rather than placing technical governance on individual clinicians.

### Mixed-Fault Scenarios and Layered Accountability in Practice

The central discussion is not whether AI should be used in psychiatry, but how responsibility should be allocated when AI becomes part of psychiatric judgment. A layered accountability model is preferable because it matches the sociotechnical structure of AI-assisted care. Developers primarily control design, validation, performance claims, technical documentation, cybersecurity, safeguards, and foreseeable misuse. Deployers primarily control procurement, local validation, workflow, training, escalation, monitoring, and audit. Clinicians control contextual interpretation, explanation to the patient, assessment of clinical coherence, and documentation of reliance or departure. These boundaries are functional rather than absolute, and mixed-fault cases may involve more than one level. This model avoids treating the psychiatrist as the default bearer of all digital risk, while preserving the clinician’s duty to reason with the patient rather than through the machine. Such a model also clarifies the relationship between law and ethics. The law must determine fault, causation, defect, disclosure, and compensation after harm. Ethics must preserve the conditions under which psychiatric care remains a relationship of interpretation and moral responsibility. In psychiatry, these 2 tasks are closely connected. A legal framework that encourages passive deference to AI would weaken care even before litigation arises. Conversely, a framework that places all risk on clinicians may discourage useful tools or incentivize defensive documentation. A defensible European approach should therefore combine preventive regulation, product liability, national malpractice law, professional guidance, and institutional governance rather than relying on any single mechanism.

Mixed-fault scenarios are particularly important. If a hospital mandates use of an opaque suicide-risk tool without local validation, the main failure is unlikely to be the psychiatrist’s ordinary clinical judgment alone. If a vendor markets a chatbot as supportive while designing it to maximize emotional engagement among vulnerable users, the central issue may be product design, misleading claims, or foreseeable misuse. If an LLM documentation system subtly changes the evidential tone of notes, responsibility may be shared between the developer that produced insufficient safeguards, the institution that integrated the tool without audit, and the clinician who failed to verify a high-stakes record. Conversely, when a transparent, validated, locally governed tool is used as intended, the clinician remains responsible for contextual interpretation rather than mechanical obedience. Layered accountability therefore does not dilute responsibility; it locates responsibility at the level where control and preventability were greatest.

## Limitations

The selected jurisdictions are used to illustrate governance patterns, not to establish definitive national liability positions. Some national materials are policy or institutional sources rather than primary liability law, and they are treated accordingly. The field is also changing rapidly: implementation of the AI Act, including possible changes to application timetables, national supervisory structures, the Product Liability Directive, and the EHDS Regulation may alter the practical liability landscape. Judicial interpretation of the AI Act, Product Liability Directive, and national malpractice law will be decisive, so the legal conclusions remain provisional. The proposed standards are normative and require empirical evaluation of clinician reliance, documentation burden, patient outcomes, coercive effects, therapeutic trust, and feasibility across differently resourced services. The legal analysis is specifically European. In North America and other jurisdictions, sectoral regulation, product law, malpractice, and professional oversight are arranged differently; the clinical concepts may have broader relevance, but the allocation of legal duties cannot be assumed to transfer unchanged [14].

## Conclusions

AI is likely to become a durable feature of psychiatric practice in Europe, but durability should not be confused with legal or ethical maturity. This Viewpoint argues that psychiatric AI should not be treated as a straightforward extension of conventional medical software, because it may affect testimony, trust, liberty, privacy, documentation, and psychological harm. The present regulatory architecture is developed more for market access, safety governance, and compliance than for allocation of civil liability after harm. For this reason, we propose an augmented-clinician model combined with layered accountability. Clinicians remain responsible for reasoned, patient-centered judgment; developers for design, validation, and defect; and deployers for procurement, training, workflow, and postmarket monitoring. The proposed standard of care is justified integration rather than routine deference to automation. This framework is not presented as an established European legal rule. It is offered as a clinically grounded and legally plausible direction for professional guidance, institutional governance, and future liability analysis.

## Abbreviations

AESIA: Spanish Agency for the Supervision of Artificial Intelligence
ANACOM: Autoridade Nacional de Comunicações
EHDS: European Health Data Space
EU: European Union
GDPR: General Data Protection Regulation
LLM: large language model
MDCG: Medical Device Coordination Group
MDR: Medical Device Regulation
